# Indicanuria, Its Aetiology and Practical Significance

**Published:** 1907-08

**Authors:** 


### INDICANURIA, ITS AETIOLOGY AND PRACTICAL SIGNIFICANCE.

W. H. Porter, of New York, sums up our knowledge of indicanuria as follows: It is one of the most important conditions in connection with clinical medicine, and is always the result of putrefactive fermentation. Animal proteids are more likely to undergo putrefactive fermentation than the vegetable class. Vegetable proteids are much more difficult of digestion than are the animal class, hence they are less economic, and are often detrimental to the system. Bacterial action is required to produce putrefactive fermentation in connection with the formation of indoxyl potassium sulphate comes from the proteid molecule as the result of its oxidation reduction, and indican is primarily formed in the intestinal tract, and not in the liver, while numerous toxines are formed at the same time when the indican is produced. These toxines are absorbed into the circulation from the alimentary tract, and by their action upon the nervous system excite an almost endless variety of symptoms. The conditions favoring the production of indican are errors in diet, lack of outdoor exercise, defective digestive secretions, and profound disturbances in the working of the nervous mechanism. Indican in the urine is never normal, but indicates an abnormal condition, because a putrefactive process can never be regarded as a normal or physiological phenomenon.

Successful treatment of these conditions depends absolutely upon an accurate apprehension of the ætiological factors entering into the production of indicanuria, and also the best methods for the removal of those factors.—Med. Rec. June 15th, 1907.

